# Red cell distribution width associated with adverse cardiovascular outcomes in patients with chronic kidney disease

**DOI:** 10.1186/s12882-017-0766-4

**Published:** 2017-12-13

**Authors:** Yueh-An Lu, Pei-Chun Fan, Cheng-Chia Lee, Victor Chien-Chia Wu, Ya-Chung Tian, Chih-Wei Yang, Yung-Chang Chen, Chih-Hsiang Chang

**Affiliations:** 1 Department of Nephrology, Kidney Research Center, Chang Gung Memorial Hospital, Linkou Medical Center, Chang Gung University, No. 5 Fusing Street, Gueishan Dist, Taoyuan City, 333 Taiwan, Republic of China; 2 Graduate Institute of Clinical Medical Sciences, College of Medicine, Chang Gung University, No.259, Wenhua 1st Rd., Guishan Dist, Taoyuan City, 33302 Taiwan, Republic of China; 3 Department of Cardiology, Chang Gung Memorial Hospital, Keelung Branch, No. 222, Maijin Rd., Anle Dist, Keelung City, 20401 Taiwan, Republic of China

**Keywords:** Red cell distribution width, Cardiovascular outcomes, Chronic kidney disease

## Abstract

**Background:**

Recent studies have demonstrated that red cell distribution width (RDW) is associated with cardiovascular (CV) events and mortality. Patients with chronic kidney disease (CKD) are often anemic and have high RDW levels. In this study, we investigated the effect of RDW on major composite CV outcomes among patients with CKD.

**Methods:**

We retrospectively analyzed patients with CKD who were admitted to the department of cardiology of a tertiary hospital in 2011. The patients were divided into 2 groups: normal RDW (RDW < 14.5%) and elevated RDW (RDW ≥ 14.5%). Demographic characteristics, comorbidities, blood investigation results, prescriptions, and outcomes were analyzed after a 3-year follow-up period. Six adjustment levels were performed to evaluate the effect of RDW on outcomes.

**Results:**

This study involved 282 patients with CKD: 213 in the elevated RDW group and 69 in the normal RDW group. The elevated RDW group had older patients, a lower proportion of male patients, lower left ventricular ejection fraction (LVEF) values, lower hemoglobin levels, lower serum albumin levels, and higher creatinine levels, compared with the normal RDW group. A linear trend was observed toward higher RDW in patients with deteriorating renal function. In the final adjusted model, RDW ≥ 14.5%, older age, and lower LVEF were associated with an increased risk of major composite CV outcomes.

**Conclusion:**

RDW is a potentially useful cost-effective indicator of major composite CV outcomes in patients with CKD.

## Background

Red cell distribution width (RDW), a coefficient of variation in red blood cell (RBC) volume, is routinely available in reports of complete blood count. RDW is commonly increased because of erythropoiesis deficits or accelerated RBC destruction. RDW increase is often considered as an indicator of anemia caused by iron, cobalamin, or folate deficiency [1, 2]. Recent studies have indicated that patients with higher RDW levels were associated with an increased risk of cardiovascular (CV) events and new-onset stroke [3–5]. Moreover, the level of RDW was positively correlated with mortality in patients with acute or critical illnesses, in those with chronic diseases, and in healthy cohorts [6–13]. Patel et al. reported a 14% increased risk of death for each 1% increment in RDW in a community-based elderly population [10].

Chronic kidney disease (CKD) and anemia are well-known risk factors for CV events and mortality. RDW significantly increases when CKD progresses from stage 1 to stage 5 [14]. According to our review of the relevant literature, few reports have focused on RDW in patients with CKD. Hsieh et al. revealed that RDW was an independent predictor of all-cause mortality, cardiovascular mortality, and infection mortality in CKD stages 3–5 [15]. Inadequate erythropoietin production is the primary feature of anemia in patients with CKD. Anemia reduces oxygen delivery and increases cardiac workload, which exacerbates heart failure and increases the risk of CV events. However, anemia is not the only possible explanation of elevated RDW in patients with CKD. The association of RDW with CV events and mortality was observed to be significant after adjustment for hemoglobin [16]. In this study, we investigated the effect of RDW on major composite CV outcomes during CKD stages 1–5. We hypothesized that elevated RDW is an independent risk factor for CV events and mortality in patients with CKD.

## Methods

### Study population

This study was conducted by retrospectively analyzing consecutive patients who were admitted to the Department of Cardiology at Chang Gung Memorial Hospital (a tertiary medical center) in Taiwan, from January 2011 to December 2011. Patients with CKD who were hospitalized for coronary artery disease (CAD) were enrolled on the date of admission. CKD was diagnosed using the existing anatomical abnormality of the kidney, persistent proteinuria, hematuria, or an estimated glomerular filtration rate of <90 mL/min/1.73 m^2^ for more than 3 months. We excluded patients who are aged <20 years and those who had end-stage renal disease on long-term renal replacement therapy. The Institutional Review Board at Linkou Chang Gung Memorial Hospital approved this study and waived the requirement for informed consent.

### Data collection

Normal RDW was defined as a level of 11.5%–14.5%. Patients were divided into normal RDW (RDW < 14.5%) and elevated RDW (RDW ≥ 14.5%) groups. We retrieved their demographic characteristics; comorbidities; blood investigation results; and prescriptions of antiplatelet agents, angiotensin converting enzyme inhibitor/angiotensin II receptor blockers, beta blockers, and statins. Patients were followed up for 3 years. The primary endpoint of this study was to elucidate the effect of RDW on mortality and major composite CV events, defined as re-admission for CAD or congestive heart failure during the follow-up period. The secondary endpoints were the identification of independent predictors of major composite CV outcomes in patients with CKD and determination of the variation in RDW among different CKD stages. The follow-up ended when a major composite CV event or death occurred, or when the patient remained event free till 3 years.

### Statistical analysis

Data are presented as mean ± standard deviation for continuous data and number (percentage) for categorical data. The 2 groups (RDW < 14.5% vs RDW ≥ 14.5%) were compared using a 2-sample *t* test for continuous variables and the Fisher exact test for categorical variables. The linear trend of RDW across groups of CKD stages was evaluated using a linear contrast test in a general linear model.

We used different multivariable Cox proportional hazard models to evaluate the association between RDW and primary outcomes. Various confounding factors, including demographic variables, comorbidities, and serum lipids, were further adjusted in the models. In addition to adjusting for variables related to the Framingham cardiovascular risk score, we adjusted for baseline characteristics that presented a significant difference between the 2 groups in the multivariable models (*P* < .05). The following models were used: (1) unadjusted model; (2) model adjusted for age and sex; (3) model further adjusted for diabetes mellitus (DM), hypertension (HTN), and smoking; (4) model further adjusted for total cholesterol, high-density lipoprotein (HDL), and systolic blood pressure (BP); (5) model further adjusted for hemoglobin and albumin; and (6) model further adjusted for creatinine and left ventricular ejection fraction (LVEF).

The cumulative survival curves for major composite CV outcomes during the 3-year follow-up period were generated using the Kaplan–Meier method, whereas the difference between the 2 groups was evaluated using a log-rank test. A *P* value of <.05 was considered statistically significant. This study used IBM SPSS 22 (IBM SPSS, Armonk, NY, USA: IBM Corp) for data analysis.

## Results

A total of 282 patients with CKD who were admitted for CAD in 2011 were enrolled in this study. Patients were divided into normal RDW (RDW < 14.5%; *n* = 213) and elevated RDW (RDW ≥ 14.5%, *n* = 69) groups. The baseline characteristics of the study cohort are presented in Table 1. The elevated RDW group had older patients than did the normal RDW group (68.2 ± 13.1 vs 63.7 ± 13.8, *P* = .018) and also had a lower proportion of male patients (62.3% vs 77.9%, *P* = .017). Moreover, patients in the elevated RDW group presented significantly lower left ventricular ejection fraction (LVEF; 48.4 ± 18.4 vs 55.6 ± 15.7, *P* = .017), lower hemoglobin levels (11.3 ± 2.1 vs 13.4 ± 2.3, *P* < .001), lower serum albumin levels (3.6 ± 0.5 vs 3.8 ± 0.5, *P* = .005), and higher creatinine levels (1.89 ± 1.58 vs 1.31 ± 0.93, *P* < .001), compared with those in the normal RDW group. The baseline characteristics of the 2 groups were similar in terms of comorbidities, systolic and diastolic BP, leukocyte count, platelet count, alanine aminotransferase levels, sodium levels, potassium levels, sugar levels, total cholesterol levels, low-density lipoprotein levels, HDL levels, and medications. During the 3-year follow-up period, the elevated RDW group exhibited a higher incidence of major composite CV outcomes (46.4% vs 17.8%, *P* < .001). No statistical difference was observed; however, a trend toward higher mortality was noted in the elevated RDW group (10.1% vs 3.8%, *P* = .059).

**Table 1:** Baseline characteristics of patients according to RDW

Characteristics	RDW < 14.5% (*n* = 213)	RDW ≥ 14.5% (*n* = 69)	*P* ^‡^
Age, year	63.7 ± 13.8	68.2 ± 13.1	0.018
Male gender, n (%)	166 (77.9)	43 (62.3)	0.017
Diabetes mellitus, n (%)	85 (39.9)	35 (50.7)	0.125
Hypertension, n (%)	135 (63.4)	43 (62.3)	0.887
Old CVA, n (%)	21 (9.9)	5 (7.2)	0.636
Smoker, n (%)	80 (37.6)	34 (49.3)	0.092
Systolic BP, mmHg	125.2 ± 25.6	129.0 ± 29.2	0.293
Diastolic BP, mmHg	70.3 ± 15.6	66.2 ± 14.4	0.056
LVEF, %	55.6 ± 15.7	48.4 ± 18.4	0.002
Lab data			
Leukocyte count, 1000/ml	9.9 ± 3.3	9.6 ± 3.0	0.492
Hemoglobin, g/dl	13.4 ± 2.3	11.3 ± 2.1	<0.001
Platelet count, 1000/ml	209.6 ± 60.3	207.3 ± 84.0	0.811
ALT, u/l	40.5 ± 41.5	32.8 ± 44.0	0.185
Albumin, mg/dl	3.8 ± 0.5	3.6 ± 0.5	0.005
Creatinine, mg/dl	1.31 ± 0.93	1.89 ± 1.58	<0.001
Na, mmol/L	139.4 ± 3.3	138.8 ± 3.5	0.212
K, mmol/L	3.9 ± 0.5	4.0 ± 0.5	0.416
Sugar, mg/dl	162.6 ± 77.5	155.0 ± 73.0	0.477
Total cholesterol, mg/dL	197.1 ± 37.5	198.9 ± 37.9	0.721
LDL, mg/dL	109.5 ± 32.4	111.6 ± 31.3	0.649
HDL, mg/dL	42.7 ± 12.7	46.1 ± 16.0	0.068
Medications, n (%)			
Anti-platelet agents	207 (97.2)	66 (95.7)	0.461
Beta-blocker	202 (94.8)	63 (91.3)	0.380
ACEi/ARB	182 (85.4)	54 (78.3)	0.189
Statin	196 (92.0)	62 (89.9)	0.621
Outcomes, n (%)			
Mortality	8 (3.8)	7 (10.1)	0.059
Major composite CV outcome	38 (17.8)	32 (46.4)	<0.001

*RDW* red cell distribution width, *CVA* cerebrovascular accident, *BP* blood pressure, *LEVF* left ventricular ejection fraction, *ALT* alanine aminotransferase, *LDL* low-density lipoprotein, *HDL* high-density lipoprotein, *ACEi* angiotensin converting enzyme inhibitor, *ARB* angiotensin II receptor blocker, *CV* cardiovascular

^‡^Continuous variables were compared using a *t* test and categorical variables were compared using the Fisher exact test

**Fig. 1:**
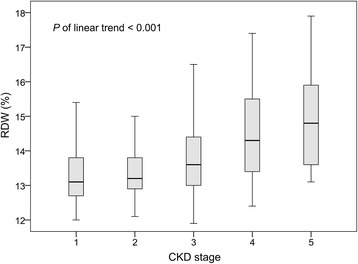
RDW levels in groups across CKD stages

We analyzed the RDW levels across CKD stages (Fig. 1). RDW increased when renal function deteriorated from CKD stage 1 to stage 5. We also observed a linear trend toward higher RDW after stratifying patients according to their CKD stage (*P* for linear trend < .001).

Table 2 presents the associations between RDW and the risk of major composite CV outcomes during the 3-year follow-up period in the 6 models. In the unadjusted model, RDW ≥ 14.5% was associated with an increased risk of major composite CV outcomes (Hazard ratio [HR], 3.22, 95% confidence interval [CI], 2.01–5.15). We first adjusted the variables of the Framingham CV risk score, including age, sex, diabetes, HTN, smoking, total cholesterol, HDL, and systolic BP. The association was significant after adjustment for age and sex (model 2: HR, 3.15; 95% CI, 1.94–5.12); further adjustment for DM, HTN, and smoking (model 3: HR, 3.14; 95% CI, 1.92–5.13); and further adjustment for total cholesterol levels, HDL levels, and systolic BP (model 4: HR, 3.25; 95% CI, 1.99–5.31). We then adjusted for demographic variables that exhibited a significant difference (Table 1): hemoglobin, albumin, creatinine, and LVEF levels in models 5 and 6. Despite the adjustments for hemoglobin and albumin levels (model 5: HR, 2.83; 95% CI, 1.70–4.70) and further adjustment for creatinine and LVEF levels (model 6: HR, 2.58; 95% CI, 1.54–4.32), RDW ≥ 14.5% was independently associated with a higher risk of major composite CV outcomes in patients with CKD.

**Table 2:** Association between RDW and risk of major composite cardiovascular outcomes in various adjustment models

Model	Presence of RDW ≥ 14.5%		
	HR	95% CI of HR	*P* value
Model 1, unadjusted model	3.22	2.01–5.15	<0.001
Model 2, adjusted for age, gender	3.15	1.94–5.12	<0.001
Model 3, further adjusted for diabetes mellitus, hypertension, smoking	3.14	1.92–5.13	<0.001
Model 4, further adjusted for total cholesterol, HDL, Systolic BP	3.25	1.99–5.31	<0.001
Model 5, further adjusted for hemoglobin, albumin	2.83	1.70–4.70	<0.001
Model 6, further adjusted for creatinine, LVEF	2.58	1.54–4.32	<0.001

*RDW* red cell distribution width, *HR* hazard ratio, *CI* confidence interval, *HDL* high-density lipoprotein, *BP* blood pressure, *LEVF* left ventricular ejection fraction

**Fig. 2:**
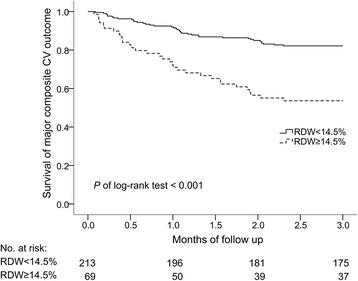
Kaplan–Meier survival curves of major composite CV outcomes during a 3-year follow-up period according to RDW levels

Major composite CV events developed in 32 of the 69 patients in the RDW ≥ 14.5% group (46.4%) and 38 of 213 patients (17.8%) in the elevated RDW group during the 3-year follow-up period. The Kaplan–Meier survival curves of major composite CV outcomes according to RDW status are presented in Fig. 2. Patients in the elevated RDW group exhibited significantly lower event-free survival (*P* value of log rank test < .001).

In the final multivariable model (model 6 in Table 2), RDW ≥ 14.5% (HR, 2.581; 95% CI, 1.541–4.322) and older age (HR, 1.022; 95% CI, 1.001–1.043) were associated with an increased risk of major composite CV outcomes (Table 3). However, higher LVEF values were associated with a lower CV risk (HR, 0.975; 95% CI, 0.961–0.989).

**Table 3:** Factors associated with major composite CV outcomes during a 3-year follow-up period

Variable	HR	95% CI of HR	*P* value
RDW ≥ 14.5%	2.581	1.541–4.322	<0.001
Age (year)	1.022	1.001–1.043	0.037
Male gender	1.587	0.849–2.964	0.148
Diabetes mellitus	0.971	0.580–1.627	0.911
Hypertension	1.004	0.566–1.781	0.989
Smoking	0.907	0.552–1.491	0.701
Total cholesterol (mg/dL)	1.001	0.995–1.007	0.683
HDL (mg/dL)	0.987	0.969–1.006	0.174
Systolic BP (mmHg)	1.001	0.991–1.011	0.873
Hemoglobin (g/dl)	0.941	0.823–1.076	0.373
Albumin (mg/dl)	0.844	0.489–1.459	0.545
Creatinine (mg/dl)	1.113	0.914–1.355	0.286
LVEF, %	0.975	0.961–0.989	0.001

*CV* cardiovascular, *HR* hazard ratio, *CI* confidence interval, *RDW* red cell distribution width, *HDL* high-density lipoprotein, *BP* blood pressure, *LEVF* left ventricular ejection fraction

## Discussion

In this study, we investigated the association of RDW with major composite CV outcomes and mortality in patients with CKD. The elevated RDW group had older patients, a lower proportion of male patients, lower LVEF levels, lower hemoglobin levels, lower albumin levels, and higher serum creatinine levels compared with the normal RDW group. RDW levels exhibited a graded increase across different stages of CKD. RDW ≥ 14.5% was associated with a significantly higher incidence of major composite CV outcomes and a trend toward higher mortality. A greater than 2-fold risk of major composite CV outcomes was observed in the elevated RDW group (HR = 2.581, CI = 1.541–4.322, *P* < .001). Age and impaired LVEF were additional covariates that were independent predictors of major composite CV outcomes in patients with CKD. The incidence of outcomes is higher than that reported in a previous study, because our patients were confirmed to have CAD [15].

Patients with higher RDW levels are often considered to have erythropoietic stress. In the general population, patients with higher RDW levels are likely to be older and have smoking habits, higher body mass index, lower hemoglobin levels, lower mean corpuscular volume, and higher serum creatinine levels [12, 16, 17]. Elevated RDW was correlated with higher serum levels of N-terminal pro-B-type natriuretic peptide (NT-proBNP), a larger left atrium, and lower LVEF values in patients with CV risk [5, 18]. RDW levels were also positively associated with C-reactive protein, the erythrocyte sedimentation rate, ferritin, and fibrinogen and negatively associated with albumin levels [6, 12, 14, 17]. Consequently, RDW was supposed to be a marker of inflammation, oxidative stress, and nutritional status [9, 19].

The pathophysiology of elevated RDW toward unfavorable CV outcomes has not been elucidated. Anemia and elevated serum creatinine, commonly occurring in patients with CKD, were risk factors for CV events. Our results reveal that the effect of RDW on major composite CV outcomes was independent of hemoglobin and creatinine levels. This finding is consistent with those of previous studies, which have reported that the level of RDW, even within the normal reference range, was associated with a higher risk of CV events and mortality irrespective of anemia, ferritin levels, and renal impairment [9, 10, 13–17]. There should be other explanations for the poor prognostic effect of RDW on major composite CV outcomes in patients with CKD. Two novel risk factors for CV events, oxidative stress and inflammation, could be possible underlying mechanisms.

Oxidative stress and inflammation suppress erythropoiesis and shorten RBC survival, resulting in heterogeneity in the size of circulating erythrocytes (also called anisocytosis). Human erythrocyte damage starts during the initial stages of oxidative stress [20]. A low level of serum selenium, an essential trace element involved in the antioxidant system, was an independent predictor of higher RDW in community-dwelling adults [21]. Serum interleukin-6 (IL-6), a proinflammatory cytokine, was positively correlated with RDW in adult patients with heart failure and congenital heart disease [22, 23]. IL-6 stimulates hepatic hepcidin expression, which inhibits duodenal iron absorption and induces ferritin transcription, thus increasing iron storage within macrophages [24]. Inflammatory cytokines such as interferon-γ, tumor necrosis factor-α, interleukin-1, and interleukin-10 might compromise iron metabolism, impair erythroid progenitor cell proliferation, and blunt erythropoietin response to anemia [24]. We supposed that elevated RDW could reflect oxidative and inflammatory stress, which is correlated with the risk of CV events and mortality [12, 23]. In a small randomized trial of patients undergoing hemodialysis, the use of a vitamin E-bonded cellulose membrane dialyzer, which was supposed to provide an antioxidant effect, reduced RDW levels and improved atherosclerosis [25]. This hypothesis must be investigated further in the future.

Recent studies have demonstrated that elevated RDW was associated with renin–angiotensin–aldosterone system (RAAS) activation, autonomic regulation, malnutrition, and endothelial dysfunction. Erythropoiesis has been proven to be associated with chronic activation of the RAAS and could be influenced by catecholamine [26–29]. Vashistha et al. reported that for every 1 g/dL increase in the albumin level, RDW decreased by 0.7%. Solak et al. revealed that a higher RDW level was independently associated with impaired endothelial function and increased carotid intima media thickness in patients with CKD [14]. In addition, RDW was associated with an abnormal pattern of circadian BP variation. RDW was higher in patients designated as nondippers compared with those designated as dippers [30]. These characters are well-described risk factors for CV events and might be possible pathways underlying the association between RDW and CV outcomes. Additional studies are warranted to verify these potential mechanisms.

The association between RDW and mortality was linear and consistent across various RDW levels, ranging from the normal reference level to RDW ≥ 17.5% [6, 16]. We demonstrate the prognostic significance of RDW in this study. Because biomarkers of oxidative stress and inflammation such as myeloperoxidase, IL-6, and interferon-γ are not routinely available in general practice, RDW might be a cost-effective tool for physicians for identifying populations at high CV risks. However, similar to other markers of infection or inflammation, RDW is not specific for a particular disease. Therefore, the feasibility of its use in clinical applications remains unclear.

Some limitations of our study must be discussed. First, because we retrospectively analyzed our study cohort, we could determine only the association between increased RDW and CV outcomes but not the causality. Second, the study was conducted at a single center and included a relatively small number of patients; hence, our sample might not reflect the entire cohort. Third, although we controlled for several variables influencing RDW, there might be relevant confounders that we were unable to completely adjust. A well-designed prospective study with a larger cohort may be performed to verify our results.

## Conclusions

In patients with CKD, a linear trend toward higher RDW was observed with the progress of the disease. Our results suggest RDW to be an indicator of CV events in patients with CKD during a 3-year follow-up period. Moreover, old age and decreased LVEF were additional risk factors for CV events. Oxidative stress and inflammation could be possible pathways underlying the association between RDW and unfavorable CV outcomes. Moreover, RAAS activation, autonomic regulation, malnutrition, and endothelial dysfunction might be potential mechanisms.

## Abbreviations

BP: Blood pressure
CAD: Coronary artery disease
CI: Confidence interval
CKD: Chronic kidney disease
CV: Cardiovascular
DM: Diabetes mellitus
HDL: High-density lipoprotein
HR: Hazard ratio
HTN: Hypertension
IL-6: Interleukin 6
LVEF: Left ventricular ejection fraction
NT-proBNP: N-terminal pro-B-type natriuretic peptide
RAAS: Renin–angiotensin–aldosterone system
RBC: Red blood cell
RDW: Red cell distribution width
